# The effects of mifepristone on the structure of human decidua and chorion and Bax and Bcl-2 expression at early stage of pregnancy

**DOI:** 10.1186/s40360-022-00592-4

**Published:** 2022-07-23

**Authors:** Fei Tian, Hua Han, Ligang Jia, Junqin Zhang, Zhaoping Chu, Jie Li, Yuan Zhang, Ping Yan

**Affiliations:** Department of Gynaecology, Hebei General Hospital, 348 Heping West Road, Shijiazhuang, 050051 Hebei China

**Keywords:** Mifepristone, Decidua and chorion, Histology, Pregnancy, Apoptosis

## Abstract

**Background:**

As a progesterone receptor antagonist, mifepristone combined with misoprostol is widely used to terminate early pregnancy in clinical practice. It has also been reported that mifepristone may cause cell death in decidual cells and result in hemorrhage of the decidua and insufficient blood supply. However, little is known about the histological effects of mifepristone on human decidua and chorion.

**Methods:**

Histological and subcellular structural changes of decidua and chorionic villi from women taking mifepristone at early pregnancy times were examined by Hematoxylin and eosin (H&E) staining and transmission Electron microscope. The expression of apoptosis-related proteins Bax/Bcl-2 was examined by immunohistochemistry.

**Results:**

After 48 h of mifepristone administration, the decidua tissue and chorionic villus structures were altered in women within 39–49 days of gestation and displayed varying degrees of degeneration and necrosis-like features. Apoptotic events were observed in the decidua and chorionic villi of early pregnancy, and mifepristone treatment significantly increases the number of apoptotic cells. The increased apoptotic events were concomitant with the increased expression of Bax and decreased expression of Bcl-2.

**Conclusion:**

This study provides evidence that mifepristone induces histological and subcellular changes in decidua and chorionic villi. Mifepristone modulates the relative ratio of Bax/Bcl-2 and the increased apoptosis contributes to the pregnancy termination at early stage of pregnancy.

## Introduction

Mifepristone (RU486) is a norethindrone derivative synthesized by Roussel-Uclaf Pharmaceutical in France in 1980, with a chemical name as 11β-[4(-N,N-dimethylamino)] phenyl-17β-hydroxy- 17α(-1-propynyl)-estrogen-4,9-dien-3-one [1]. It was first proved in 1985 that mifepristone combined with prostaglandin can effectively terminate early pregnancy, making drug-mediated abortion possible [2]. The pharmaceutical application of mifepristone is not only limited to the termination of early pregnancy, mifepristone has been widely used in contraception and the treatment of hormone-dependent diseases, such as endometriosis, adenomyosis, uterine fibroids, endometrial cancer and ovarian cancer [3–5]. It has become one of the important drugs for the treatment of obstetrics and gynecology diseases.

In recent years, extensive research has been focusing on the mechanisms of action and clinical applications of mifepristone [6, 7]. Studies suggest that mifepristone terminates early pregnancy through a variety of mechanisms of action. Mifepristone competitively inhibits progesterone by binding to the progesterone receptor in the decidua, causing degeneration and necrosis of the decidua tissue [8, 9]. On the other hand, by promoting the apoptosis of decidual cells, it leads to hemorrhage of the decidua and insufficient blood supply [10, 11]. In addition, mifepristone promotes uterine contraction and increases cervical collagen decomposition, which induces cervical maturity and cervical dilation to terminate early pregnancy [12, 13]. However, little is known about the histological effects of mifepristone on human decidua and chorion.

In this study, we investigated the histological structural changes of decidua and villi from women taking mifepristone at different early pregnancy times, as well as and the expression of apoptosis-related proteins Bax/Bcl-2. We further discussed the effects and mechanisms of mifepristone inducing apoptosis of decidua and chorionic cells during different early pregnancy times, which provides a theoretical basis for the optimal use of mifepristone in early pregnancy termination.

## Materials and methods

### Study subjects

The specimens were taken from January 2018 to October 2018 in the gynecological clinic of Hebei Provincial People's Hospital. A total of 40 women who met the following conditions and voluntarily terminated their pregnancy by medication were enrolled: (1) Women of childbearing age; (2) Normal menstrual periods, menopause ≤ 39 days, intrauterine pregnancy is confirmed by gynecological examination and B-ultrasound; (3) Fetal sac diameter < 10 mm; Fetal sac size is consistent with the gestational age; (4) No breastfeeding in the past 3 months, no hormones and anti-prostaglandin drugs within half a year; (6) No inflammation and tumor of the reproductive system; (7) No intrauterine device placed; (8) No contraindications to the use of mifepristone (9) No other chronic diseases of the system. In addition, 20 early pregnancy women with 40 days ≤ menopause ≤ 49 days were randomly selected. B-ultrasound confirmed that the size of the fetal sac was consistent with the gestational age, and the inclusion conditions were the same as above.

The 40 women who had voluntarily terminated their early pregnancy within 39 days of pregnancy were randomly divided into 2 groups: control group and the treatment group A. For the control group, the pregnancy was terminated by conventional vacuum suction. In addition, 20 early pregnant women (40 days ≤ menopause ≤ 49 days) were assigned as treatment group B. For treatment group A and B, mifepristone (150 mg) was taken once, and misoprostol (400 μg) was given vaginally after 48 h. The subjects were fasted for 2 h before and after taking the medicine, and underwent negative pressure aspiration after 2 h. At the same time, the decidua and chorionic tissues of each group were collected.

### Chemicals and reagents

Mifepristone (25 mg/tablet) and Misoprostol (200 µg/tablet) was purchased from Beijing Zizhu Pharmaceutical Co., Ltd. (Beijing, China). Mouse anti-human Bax and Bcl2 antibodies were purchased from Fuzhou Maixin Biological Co., Ltd. (Fuzhou, China). TUNEL cell apoptosis in situ detection kit was purchased from Nanjing KGI Biotechnology Development Co., Ltd. (Nanjing, China). SP9001 kit and DAB chromogenic reagent were purchased from Beijing Zhongshan Biotechnology Co., Ltd. (Beijing, China).

### Tissue section preparation

The collected decidua and chorionic villi were fixed in 10% formaldehyde. After 24 h, the fixed tissue block was dehydrated with conventional gradient of alcohol, and then processed with xylene, embedded in paraffin make a wax block. A Leica RM2125 microtome was used for continuous paraffin sectioning with a thickness of 6 μm. 10 samples of decidua and chorionic tissue were taken from each group for later use.

### Hematoxylin and eosin (H&E) staining

A total of 4 tissues samples were randomly selected from each group. The prepared sections are deparaffinized and dehydrated and then rinsed with distilled water. Samples were stained with Hematoxylin solution for 1 min and rinsed with tap water. The sections were further processed by 1% hydrochloric acid for 1 min, and then stained with Eosin solution for 2 min. The sections were dehydrated by conventional alcohol gradient and sealed with neutral gum, and observed under a light microscope.

### Transmission Electron microscope (TEM) sample preparation

The decidua and villus tissues obtained in each group were immediately washed with cold saline, and the tissues were cut into 1mm^3^ size and immediately placed into the pre-fixative solution in (4% glutaraldehyde) for 1 h at 4℃. The pre-fixed tissues were washed three times with phosphate buffered saline (PBS). The tissues were then fixed with 1% osmic acid for 2 h at 4℃. After washing, the tissues were dehydrated and infiltrated with the mixture of acetone and resin (3:1) for 15 min, and then infiltrated with pure resin for 30 min. After infiltration, samples were embedded in epoxy resin 812 and 815 embedding solution, followed by incubation in ovens at 37 °C, 45 °C, and 60 °C for 24 h respectively. The tissues were cut into ultrathin sections with a thickness of 50 nm using Leica microtome. Double electro-staining was performed with uranyl acetate for 15 min and lead citrate for 30 min. Hitachi H-7500 transmission electron microscope (accelerating voltage 80 kV) was used to observe the ultrastructure of decidua and villi in each group.

### Immunohistochemistry (TUNEL, Bax and Bcl-2 staining)

#### Slide section preparation

The decidua and villus tissues collected in each group were fixed in 4% formaldehyde for 24 h, and 10 samples were randomly selected from each group. The fixed tissue blocks were dehydrated by conventional gradient alcohol, transparent in xylene, embedded in paraffin, and made into wax blocks. A series of paraffin sections with a thickness of 6 μm were generated by microtome.

#### TUNEL staining for apoptosis

Four percent formalin-fixed and paraffin-embedded sections were deparaffinized and rehydrated. The sections were then immersed in a staining jar containing proteinase K digestion solution and digested at room temperature for 15 min. After 3 times wash in PBS, the sections were placed in 0.5% hydrogen peroxide for 20 min at room temperature. TdT enzyme reaction solution was added dropwise to cover the sample evenly and the slide was incubated in a humid box at 37 °C for 1 h, and the reaction was terminated by stop/wash solution. After drying, 50 μL of Streptavidin-HRP working solution was added to the slide for 30-min incubation at 37 °C, followed by color development using DAB chromogenic solution. The samples were dehydrated and mounted using 100% n-butanol (3 times, 2 min each time) and Xylene (3 times, 2 min each time). For negative control, TdT enzyme was not added to the samples for the reaction. Apoptotic cells were determined by the presence of brown-yellow granules in the nucleus. The mean values of the 6 fields were taken as the measurement of each sample. Apoptosis index is calculated as the number of cells with positive staining in the nucleus/total number of cells × 100%.

#### Bax/Bcl-2 immunohistochemical staining

Deparaffinized and rehydrated sections were incubated with 3% methanol-hydrogen peroxide for 10 min at room temperature to block endogenous peroxidase activity. Antigen retrieval was performed with 0.01 M citrate (PH 6.0) at 94–98 °C for 20 min. 10% normal goat serum was added dropwise to the sample to block nonspecific binding sites at 37 °C for 10 min. Primary antibody (mouse anti-human Bax (1:100), Bcl-2 (1:100) monoclonal antibody) was added dropwise to the sample for overnight incubation at 4 °C. After wash with PBS, samples were incubated with biotin-labeled secondary antibody working solution (goat anti-mouse IgG, 1:2000) for 30 min at 37 °C. After wash with PBS, horseradish peroxidase-labeled streptavidin working solution was added to the samples for incubation at 37 °C for 30 min, which was followed by DAB color development for 1–5 min. Samples were counter stained with Hematoxylin for a few seconds. The positive staining of Bax and Bcl-2 manifests as brown-yellow granules in the cells. Average optical density of positive cells in each field was measured to indicate the expression of Bax and Bcl-2 in 6 random fields (× 400 magnification).

### Statistics

The measurement data are expressed as (mean ± standard deviation), and all data were processed with SPSS13.0 statistical software. The statistical difference between two groups was compared using unpaired student’s t tests. Comparisons among multiple groups were analyzed using one-way analysis of variance (ANOVA) with Tukey’s post hoc test for pairwise comparison. *P* < 0.05 was considered to be statistically different.

## Results

### Clinical characteristics of subjects in three groups

We first examine the clinical characteristics of subjects in control, treatment A and treatment B groups There was no significant difference in age, menopause days, pregnancy/ delivery times and fetal sac size between the control and treatment A group (*P* > 0.05). Between the subjects in treatment A and B groups, there was no significant difference in age and pregnancy/ delivery times, however a significantly longer menopause days and larger size of fetal sac was found in treatment group B (*P* < 0.05) (Table 1).

**Table 1:** The clinical parameters of the different groups

Group	Control (1)	Treatment A (2)	Treatment B (3)
Subjects	20	20	20
Age	28.6 ± 5.12	29.5 ± 5.86	29.5 ± 5.26
Pregnancy times	2.5 ± 1.25	2.4 ± 1.32	2.5 ± 1.02
Delivery times	0.7 ± 0.45	0.7 ± 0.51	0.8 ± 0.55
Menopause days	37.65 ± 1.26	37.44 ± 1.53	43.05 ± 4.63
Gestational sac size	8.65 ± 3.1	8.25 ± 2.7	20.65 ± 2.9

1 vs 2: comparison of age, pregnancy, delivery times, days of menopause, and size of gestational sac, *p* > 0.05;

2 vs 3: comparison of age, pregnancy, delivery times, *p* > 0.05;

2 vs 3: comparison of days of menopause, and size of gestational sac, *p* < 0.05

### Histological changes in deciduas

**Fig. 1:**
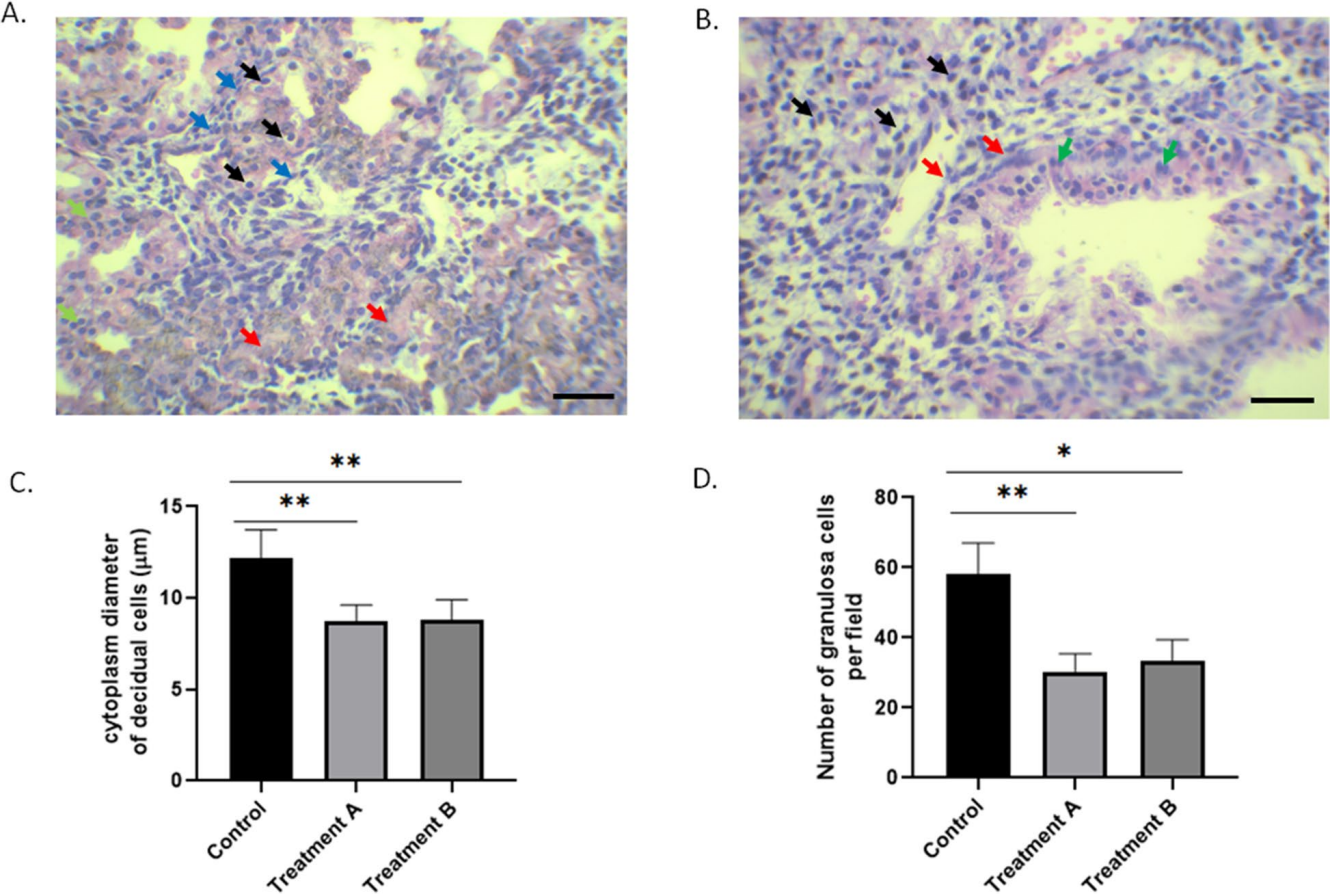
H&E staining of decidua tissues in control (**A**) and treatment A group (**B**). Scale bar: 300 µm. In figure A, decidual cells (Black arrows), granulosa cells (Blue arrows), dense reticular fibers (Red arrow) and epithelial cells (green arrow). In figure B, decidual cells (Black arrows), degenerated sheet of decidual cells (Red arrow), granulosa cells (Green arrow). **C** Quantification of the cytoplasm diameter of decidual cells in different grousp. **D** Quantification of the number of granulosa cells in different group. Data in C and D are summary of ten different slides from 5 different samples. One-way ANOVA, * < 0.01, **P < 0.01

We next examined the histological changes in decidua among three groups. The decidual tissue of the control group was composed of large decidual cells and granulosa cells. The decidual cells were polygonal or oval in shape with an oval-shaped nucleus. They are closely-arranged, with a large and transparent cytoplasm (Black arrows). The nuclei of granulosa cells are twisted (Blue arrows). The two types of cells are surrounded by dense reticular fibers (Red arrow), and the glandular epithelium is cubic or squamous epithelial cells (green arrow) (Fig. 1A).

In the decidual tissue of treatment A group, the cytoplasm volume of decidual cells decreased and the nucleus became condensed, with an increasing intercellular space (Black arrows) (Fig. 1B and C). They displayed as a focal or sheet-like cellular degeneration, which suggests potential necrotic events (Red arrow). The number of granulosa cells was decreased, with the vasodilation and damages of interstitial blood vessels (Green arrow) (Fig. 1B and D). The histological changes in experiment B group were similar as treatment A group.

### Histological changes in chorionic villi

**Fig. 2:**
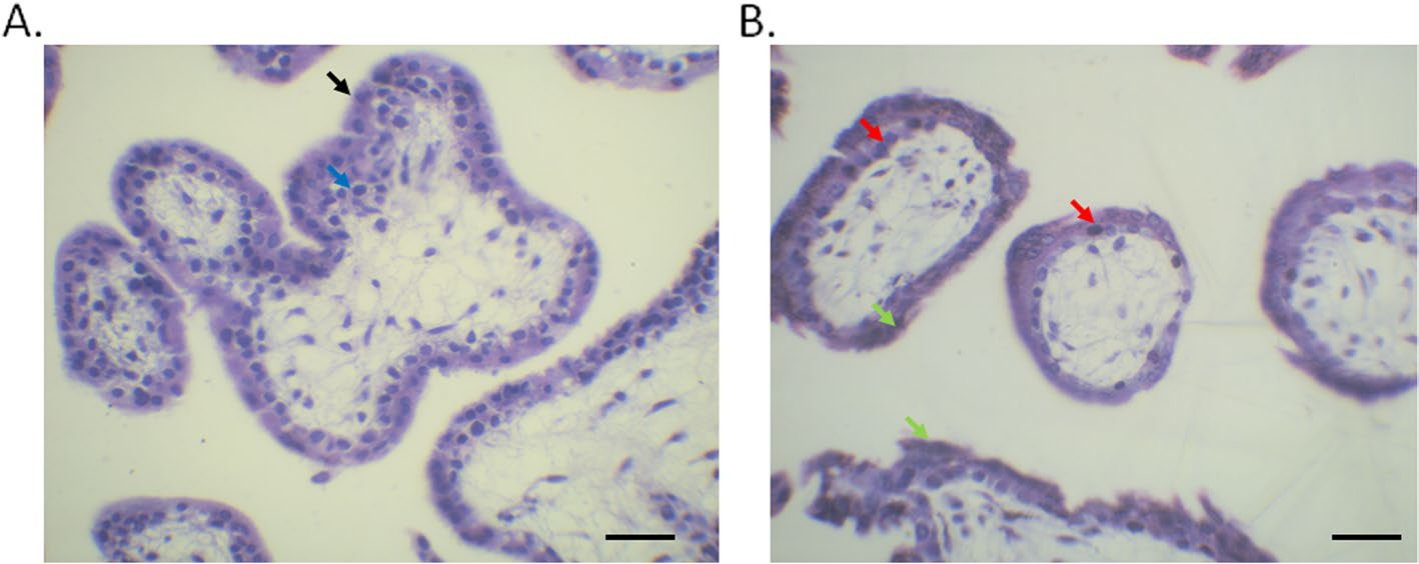
H&E staining of chorionic villi tissues in control (**A**) and treatment A group (**B**). Scale bar: 300 µm. In figure **A** syncytiotrophoblasts (Black arrow) and cytotrophoblasts (Blue arrow). In figure **B** villous trophoblast cells (Red arrow) and fibrin-like substances in the villus interstitium (Green arrow)

In the control group, the structure of villi was clear, the outer layer was syncytiotrophoblasts (Black arrow), and the inner layer was cytotrophoblasts (Blue arrow). The syncytiotrophoblast cells display dense cytoplasm and densely stained nucleus, with bristle-like cellular boundary. Cytotrophoblasts are monolayer cubic cells with clear cell boundaries, with a large and round nucleus (Fig. 2A). The villous trophoblast cells in the treatment group A are degenerated and showed necrosis-like features (Red arrow) (Fig. 2B). There is an interstitial dilatation and edema, and cells are dispersed with a condensed nucleus. Blood stasis and fibrin-like substances were observed in the villus interstitium (Green arrow). The histological changes in experiment B group were similar as treatment A group.

### Ultrastructure of decidual tissues

**Fig. 3:**
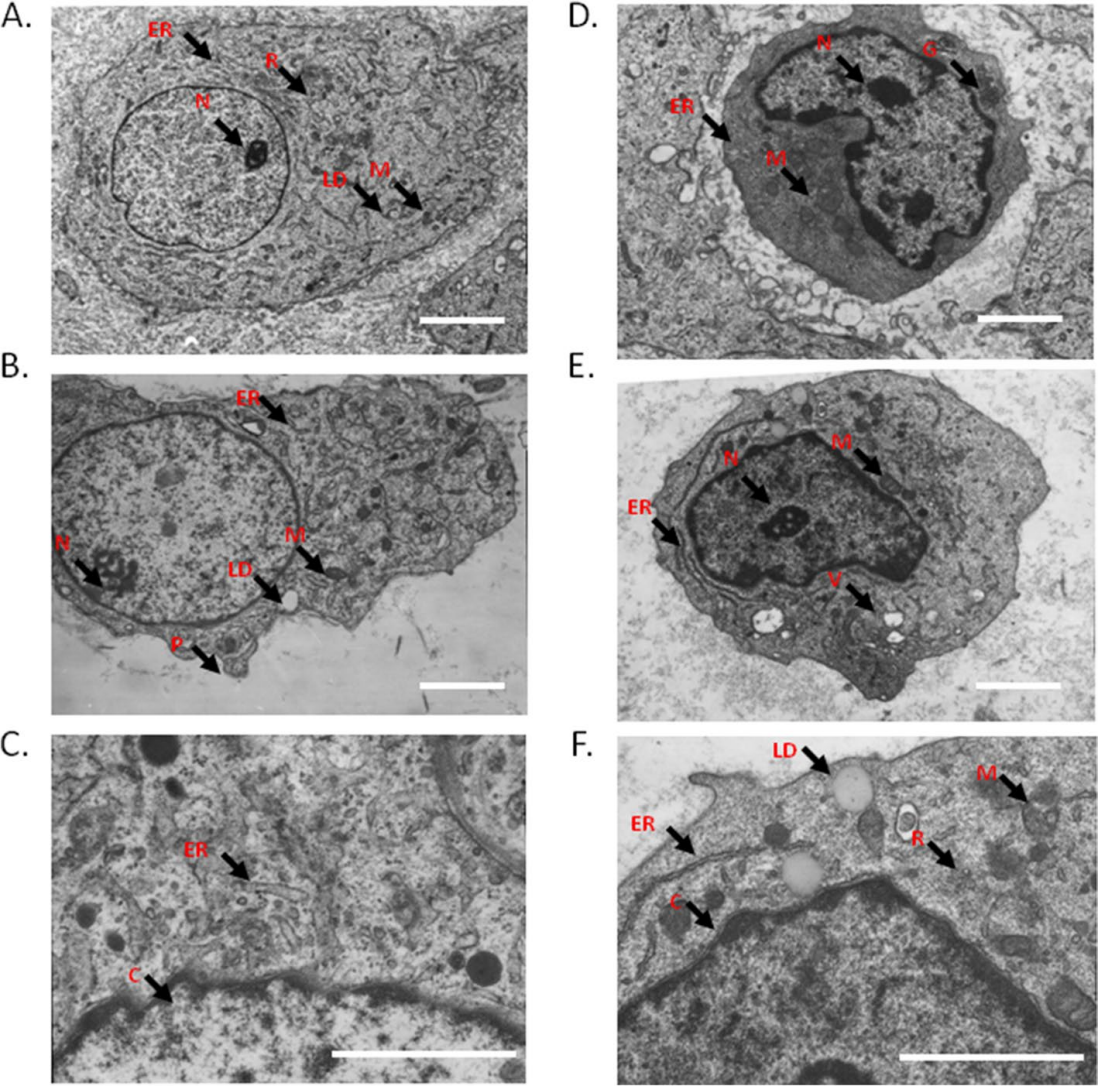
TEM images of the ultrastructure of decidual cell (**A**) and granular cells (**D**) in control group, and decidual cell (**B**-**C**) and granular cells (**E**–**F**) in treatment A group. Scale bar: 2 µm. ER: endoplasmic reticulum; M: mitochondria; C: chromatin; LD: lipid droplet; N: nucleolus; V: vacuole.; R: ribosome; P: protrusion

The decidual tissue contains two types of cells with different structural characteristics (large decidual cells and granulosa cells), which are surrounded by fine and intertwined mesh fibers. Decidual cells are larger in size, with a lower electron density, an irregular cell shape, rich and evenly distributed euchromatin in the nucleus, 1–2 large and prominent nucleoli. In the cytoplasm, there are abundant organelles including abundant mitochondria, well-developed rough endoplasmic reticulum, lipid droplets and free ribosome (Fig. 3A). Granular cells are smaller in size, with high electron density, distorted nucleus, few nucleoli, and edged heterochromatin. There are a large number of high electron density granules in the cytoplasm, and a large number of mitochondria with a large volume and cristae (Fig. 3D).

In treatment A group, the decidual cells shrink and develop increased number of pseudopod-like protrusion. The cytoplasm contains a large number of cystic-dilated rough endoplasmic reticulum and mitochondria (Fig. 3B). Most of the cristae and part of the membrane of the mitochondria are fused, with indistinct boundary. The rough endoplasmic reticulum is expanded with certain degree of degranulation. The chromatin tends to be aggregated on the edge of the nucleus (Fig. 3C). In treatment A group, the high electron density granules decrease in granulosa cells and there are vacuoles of different sizes present in the cytoplasm (Fig. 3E). There are secondary lysosomes, and enlarged lipid droplets in the cytoplasm, and rough endoplasmic reticulum expands in an irregular shape. There are more free ribosomes and mitochondria show partial cristae fusion (Fig. 3F). The changes in treatment B group were basically the same as those in group A.

### Ultrastructure of villous tissues

**Fig. 4:**
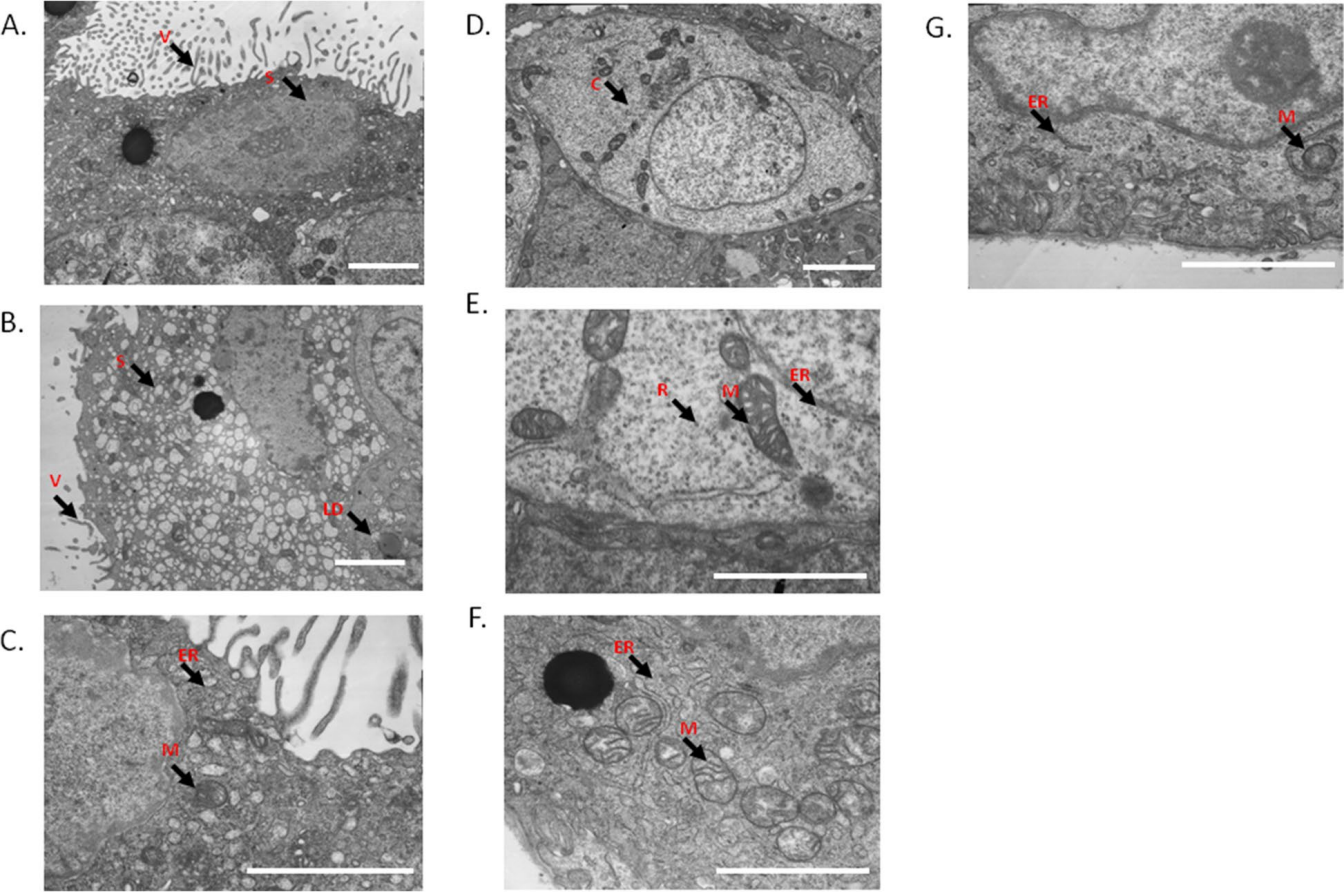
TEM images of the ultrastructure of syncytiotrophoblasts (**A**) and cytotrophoblasts (**C**-**E**) chorionic villi tissues of control group, and syncytiotrophoblasts (**B**, **F**) and cytotrophoblasts (**G**) in treatment A group. Scale bar: 2 µm. ER: endoplasmic reticulum; M: mitochondria; C: cytotrophoblasts; LD: lipid droplet; N: nucleolus; S: syncytiotrophoblasts; V: villi; R: ribosome

In the control tissues, the outer layer of chorionic villi contains syncytiotrophoblasts (Fig. 4A), cytotrophoblasts are located at the inner layer (Fig. 4D). The free border of syncytiotrophoblast cells has a large number of slender microvilli, some of which are branched, the nucleus is irregular, and the surface is smooth. The chromatin in the nucleus is evenly distributed, and the nucleoli are prominent. The cytoplasm contains a large number of rough endoplasmic reticulum and mitochondria (Fig. 4C). The cytotrophoblasts are located between the syncytiotrophoblasts and the basement membrane of the villi, and cell protrusions and desmosomes can be observed in the intercellular space. The nucleus is large with an irregular surface, and the chromatin is evenly distributed in the nucleus (Fig. 4D). There are prominent mitochondria, rough endoplasmic reticulum and free ribosome in the cytoplasm, with well-structured basement membrane (Fig. 4E).

The villous syncytiotrophoblast cells in treatment A group are found to fall off in sheets, and the number of microvilli on the surface decrease (Fig. 4B). The nuclei appear to be distorted with a curved nuclear envelope, and the nuclear chromatin is unevenly distributed. Free ribosomes are significantly reduced and cellular vacuoles increases. Partial fusion or disorganization of mitochondrial cristae is also observed (Fig. 4B and F). The cytotrophoblast cells become flat and small, the cell outline is unclear, and the nuclear chromatin distribution is sparse. Large and round lipid droplets are observed in the cytoplasm, and the rough endoplasmic reticulum is irregularly expanded. Partial fusion or disorganization of mitochondrial cristae is also observed (Fig. 4G). The changes of ultrastructure of villous tissues in treatment B are similar as treatment A group.

### Apoptosis analysis by TUNEL assay

**Fig. 5:**
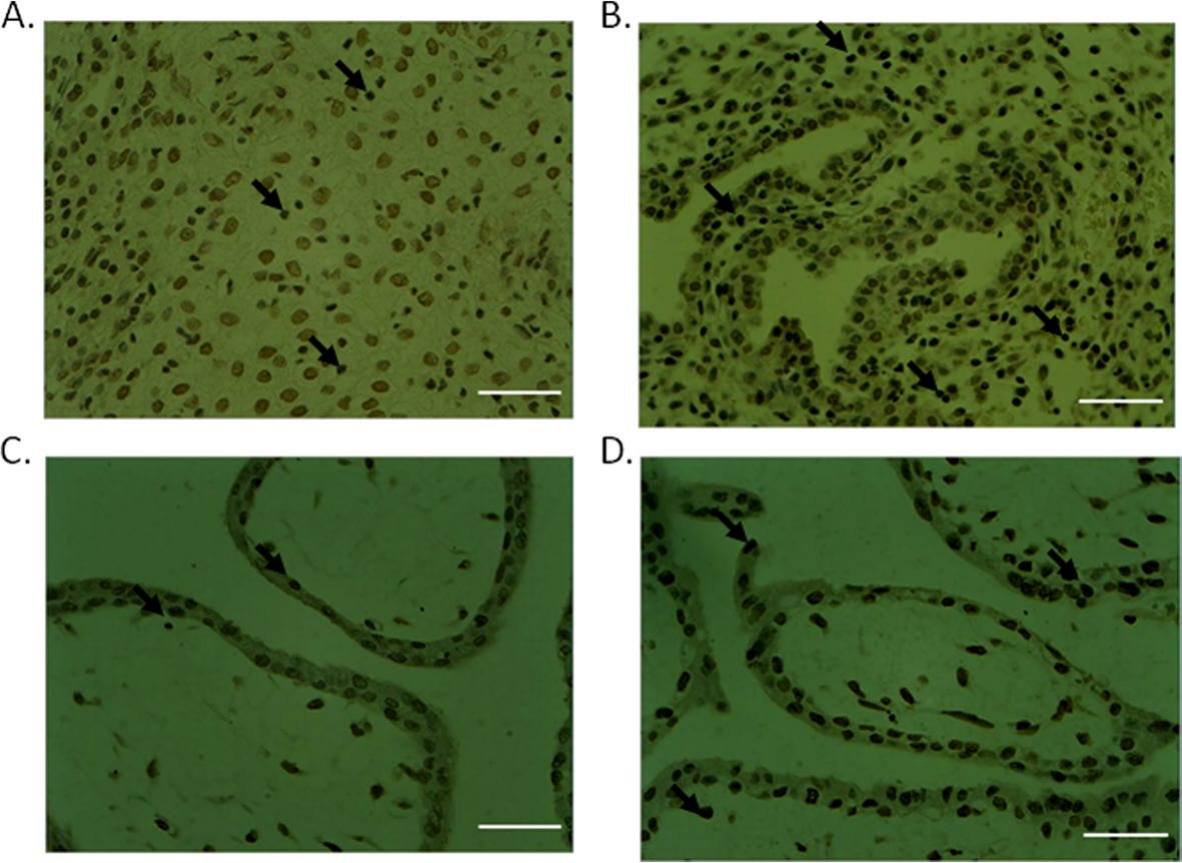
TUNEL staining of apoptotic cells in decidua tissues (**A**-**B**) and chorionic villi tissues (**C**-**D**). Control tissues: **A** and **C**; and treatment A group: **B** and **D**. Scale bar: 300 µm. Black arrows indicate apoptotic cells with string TUNEL staining signal in the nucleus

TUNEL staining revealed that the apoptotic cells stained with brownish yellow in the nucleus can be seen in the decidua tissue of the control group and the treatment group (Fig. 5A and B). The positively stained cells are significantly increased in treatment group, and apoptosis index of treatment group is significantly higher than that of the control group (Table 2). In chorionic villi tissues, similar results are observed that, the apoptotic cells are significantly increased in treatment group (Fig. 5C and D), and apoptosis index of treatment groups was higher than that of the control group (Table 3). These data indicate the increased apoptosis after mifepristone treatment.

**Table 2:** Apoptosis index in decidua of three groups

Group	Number of subjects	Apoptosis Index (%)
Control (1)	10	20.12
Treatment A (2)	10	51.86
Treatment B (3)	10	49.95

**Table 3:** Apoptosis index in chorion

Group	Number of subjects	Apoptosis Index (%)
Control (1)	10	17.78
Treatment A (2)	10	46.23
Treatment B (3)	10	44.72

Expression level of of Bax and Bcl-2 in decidua and chorionic villi.

**Fig. 6:**
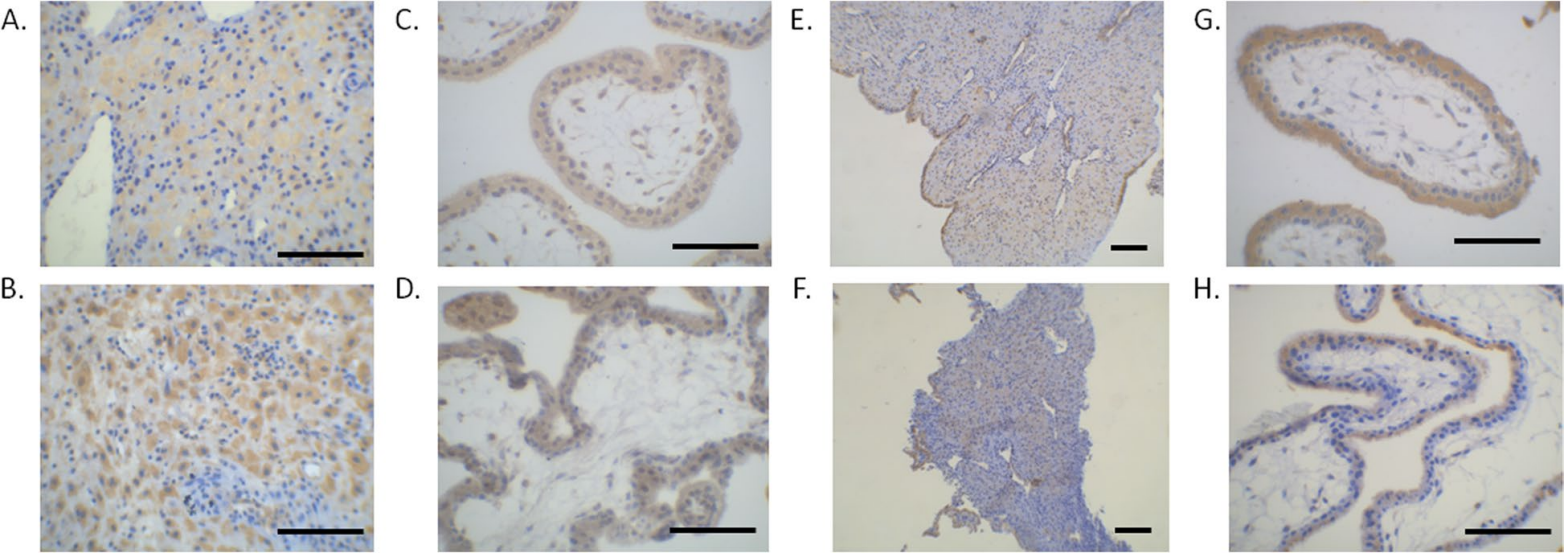
Immunohistochemical staining of Bax and Bcl-2. **A** Bax staining in decidua tissues in control group. **B** Bax staining in decidua tissues in treatment A group. **C** Bax staining in chorionic villi tissues of control group. **D** Bax staining in chorionic villi tissues of treatment A group. **E** Bcl-2 staining in decidua tissues in control group. **F** Bcl-2 staining in decidua tissues in treatment A group. **G** Bcl-2 staining in chorionic villi tissues of control group. **H** Bcl-2 staining in chorionic villi tissues of treatment A group. Scale bar: 300 µm

Cells stained positive for Bax are mainly located in the decidual stroma and decidual gland epithelium (Fig. 6A and B, the cytoplasm is stained brown). The staining of Bax-positive cells in treatment group (Fig. 6B) is denser than that in the control group (Fig. 6A). The relative Bax staining intensity in treatment group A and B is significantly higher than that of the control group, however, there is no significant difference between treatment A and B groups (Table 4). In chorionic villi, Bax staining is mainly located in the cytoplasm of the trophoblast layer in both control (Fig. 6C) and treatment group A (Fig. 6D). The relative Bax staining intensity in treatment group A and B is significantly higher than that of the control group, however, there is no significant difference between treatment A and B groups (Table 4).

**Table 4:** Relative Bax expression level in decidua and chorion

Group	Number of subjects	Bax expression level in decidua	Bax expression level in chorion
Control (1)	10	0.0865 ± 0.2767	0.1222 ± 0.1483
Treatment A (2)	10	0.1202 ± 0.0813	0.2229 ± 0.0978
Treatment B (3)	10	0.1220 ± 0.0898	0.2255 ± 0.0900

1 vs 2, *p* < 0.05; 2 vs 3, *p* > 0.05;

For Bcl-2 staining, in decidua the staining signals are mainly located in the cells of decidual stroma and decidual gland epithelium (Fig. 6E, control, and F, treatment group A). The relative Bcl-2 staining intensity in treatment group A and B is significantly lower than that of the control group, however, there is no significant difference between treatment A and B groups (Table 5). Similarly, in chorionic villi, Bcl-2 staining is mainly located in the cytoplasm of the trophoblast layer (Fig. 6G, control, and H, treatment group A) and Bcl-2 staining intensity in treatment group A and B is significantly reduced than that of the control group (Table 5). Together, these results demonstrate that mifepristone could increase the expression of pro-apoptotic protein Bax, while suppressed the level of anti-apoptotic protein Bcl-2.

**Table 5:** Relative expression level of Bcl-2 in decidua and chorion

Group	Number of subjects	Bcl-2 expression level in decidua	Bcl-2 expression level in chorion
Control (1)	10	0.2035 ± 0.0996	0.2808 ± 0.3211
Treatment A (2)	10	0.1899 ± 0.0632	0.2467 ± 0.2576
Treatment B (3)	10	0.1853 ± 0.0355	0.2259 ± 0.2624

1 vs 2, *p* < 0.05; 2 vs 3, *p* > 0.05;

## Discussion

Endometrial stroma mainly consists of two types of cells: the decidual cells are large and round, and the cytoplasm is full of lipid droplets, which function to synthesize and secrete collagen, forming reticular fibers that support the normal structure of the endometrium [14]; the granulosa cells are smaller in size and contain eosinophilic granules in the cytoplasm, which can trigger uterine contraction and cervical softening, dissolve the matrix reticular fibers in the endometrium and facilitate the shedding of inner membrane [15]. Through the histological and TEM ultrastructure analysis, we revealed that 48 h after mifepristone administration within 39 days of gestation, the cytoplasm of the large decidual cells is reduced in the decidual tissue and the nucleus is pyknotic. In granulosa cells, the long protrusions on the surface increases, the high electron density granules decrease, and there are vacuoles of different sizes present in the cytoplasm. Overall, the reticular fibers surrounding the large decidual cells and granulosa cells seem to collapse and disintegrate. These results suggest that in the decidual tissue of mifepristone-mediated abortion, the cytoplasmic endocrine granules of granulosa cells are significantly reduced and the extracellular collagen fibers are disordered and disintegrated. These changes indicate that the inhibition of the biological activity of progesterone by mifepristone results in the secretion and release of endocrine granules in granulosa cells, causing reticular fibrinolysis and rupture in decidual tissue during abortion [16, 17].

Further ultrastructure analysis of chorionic villi showed that 48 h after taking mifepristone, the villi tissue undergo dramatic changes, including degeneration and necrotic morphology of trophoblast cells, reduction in the number of surface microvilli, expansion of rough endoplasmic reticulum, and the structural degradation of intracytoplasmic organelles. These observations indicate that due to the action of mifepristone and the inhibition of progesterone, cells of the chorionic and decidual tissues show features of apoptosis and autophagy. It is commonly accepted that mifepristone terminates pregnancy by antagonizing the physiological effects of progesterone, which has multiple effects on the metabolism and morphological structure of the embryo and its supporting tissues [18, 19], including decidual cell degeneration and necrosis, decidual and villus tissue stripping, enhanced uterine contractions and apoptosis of decidual tissue in early pregnancy [20–22].

**Fig. 7:**
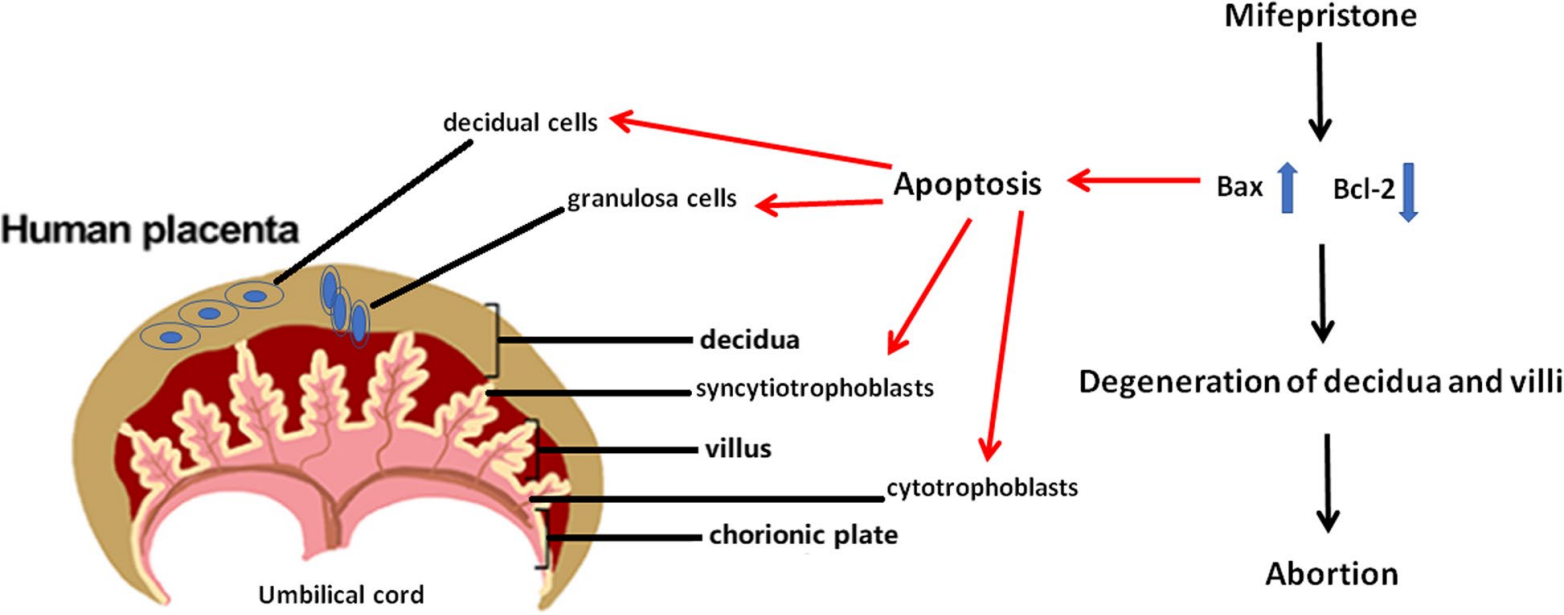
Schematics of structural changes of human decidua and chorion induced by mifepristone. Mifepristone can lead to apoptosis of cells in decidua and chorion by increasing the level of Bax and decreasing the level of Bcl-2, which eventually causes the degeneration of decidua and villi

Apoptosis is an actively regulated and programmed cell death, which is essential for embryonic development, biological metamorphosis, atrophy of endocrine organs and repair and renewal of normal tissues [23, 24]. In human placental development, trophoblast cells proliferate in large numbers; some cells differentiate and fuse to form syncytiotrophoblasts, while some syncytiotrophoblast cells undergo apoptosis [25–27]. A previous study found that administration of mifepristone to DMPA (medroxyprogesterone acetate) users significantly increases endometrial proliferation and decreases endometrial stromal apoptosis in the short term [28]. In contrast, mifepristone could inhibit cell growth and induce apoptosis in Ishikawa endometrial adenocarcinoma cells through caspase-3 activation and regulating apoptotic genes such as BCL2/BAX and FAS/FASLG [29]. The results of our study also revealed that in both normal and mifepristone-treated decidua and villus tissues, there are different degrees of apoptotic events and mifepristone treatment significantly increased apoptotic index in both decidua and chorionic tissues. Therefore, mifepristone-induced apoptosis in placental tissues may contribute to the anti-pregnancy effect (Fig. 7).

As a major anti-apoptotic protein, Bcl-2 functions to increase the resistance of cells to various apoptotic stimuli without affecting cell proliferation [30, 31]. For example, the overexpression of Bcl-2 can inhibit chemotherapy or radiotherapy-induced tumor cell apoptosis [30, 32]. Studies also showed that Bcl-2 is regularly expressed in the endometrium during menstrual cycle, which seems to regulate the apoptotic events related to cyclical changes of the endometrium [33, 34]. In contrast, Bax is one of the earliest identified pro-apoptotic protein and it promotes apoptosis by antagonizing the biological activity of Bcl-2 [35, 36]. In recent years, studies have suggested that Bcl-2 and Bax play their roles by forming homologous or heterodimers. When Bcl-2 expression level is high, it favors the formation of Bcl-2/Bax heterodimer to prevent the release of pro-apoptotic factor cytochrome C from mitochondria [35–37]. The ratio of Bcl-2/Bax seems to determine the survival of cells after the stimulation of pathogenic signals. Bax and Bcl-2 are expressed in syncytiotrophoblasts, cytotrophoblasts and decidual cells during early pregnancy, which indicates that balanced expression of Bcl-2/Bax is involved in maintaining physiological apoptosis and the reconfiguration of placental villus tissue structure [38, 39].Our data showed that the increased Bax/Bcl2 ratio after mifepristone treatment may favor the apoptotic cell death in decidual cells and the trophoblasts in the chorionic villi.

Extensive efforts have been made to investigate the mechanisms of action of mifepristone [6, 7]. Mifepristone seems to terminate early pregnancy through a variety of mechanisms of action. It competitively inhibits progesterone by binding to the progesterone receptor in the decidua, causing the degeneration of the decidua tissue [8, 9], leading to the hemorrhage of decidua [10] and resulting in insufficient blood supply [10, 11]. Mifepristone also promotes uterine contraction and increases cervical collagen decomposition [12, 13]. The histological changes and increased apoptotic events in Mifepristone-terminated chorionic villi and decidual tissues are possibly resulted from the detrimental physiological changes in the uterine endometrium. The major factors contributing to the histological changes still need to be clarified.

## Conclusion

In summary, after 48 h of mifepristone administration, the decidua tissue and chorionic villus structures are altered in women within 39 days of gestation. Decidual and villous tissues display varying degrees of degeneration and necrotic features. Apoptotic events occur in the decidua and chorionic villi of early pregnancy, and mifepristone treatment significantly increases the number of apoptotic cells. The increased apoptotic events are concomitant with the increased expression of Bax and decreased expression of Bcl-2. These data provide novel evidence that mifepristone-induced apoptosis contributes to the pregnancy termination at early stage of pregnancy.

## Data Availability

The datasets used during the present study are available from the corresponding author upon reasonable request.
